# Trends and Predictors for the Uptake of Colon Cancer Screening Using the Fecal Occult Blood Test in Spain from 2011 to 2017

**DOI:** 10.3390/ijerph17176222

**Published:** 2020-08-27

**Authors:** José Javier Zamorano-Leon, Ana López-de-Andres, Ana Álvarez-González, Clara Maestre-Miquel, Paloma Astasio-Arbiza, Antonio López-Farré, Javier de-Miguel-Diez, Rodrigo Jiménez-García, Romana Albaladejo-Vicente

**Affiliations:** 1 Department of Public Health & Maternal and Child Health, Faculty of Medicine, Universidad Complutense de Madrid, 28040 Madrid, Spain; josejzam@ucm.es (J.J.Z.-L.); pastasio@ucm.es (P.A.-A.); rodrijim@ucm.es (R.J.-G.); ralbadal.ucm@gmail.com (R.A.-V.); 2 Preventive Medicine and Public Health Teaching and Research Unit, Health Sciences Faculty, Rey Juan Carlos University, Alcorcón, 28922 Madrid, Spain; 3 Obstetrics and Gynelocogy Department, Hospital Universitario Río Ortega, 47012 Valladolid, Spain; ana-alvarez-gonzalez@hotmail.com; 4 School of Health Sciences, Universidad de Castilla la Mancha, 13071 Talavera de la Reina, Spain; clara.maestre@uclm.es; 5 Medicine Department, School of Medicine, Universidad Complutense de Madrid, 28040 Madrid, Spain; antonio.lopez.farre@med.ucm.es; 6 Respiratory Department, Hospital General Universitario Gregorio Marañón, Facultad de Medicina, Universidad Complutense de Madrid, Instituto de Investigación Sanitaria Gregorio Marañón (IiSGM), 28009 Madrid, Spain; javier.miguel@salud.madrid.org

**Keywords:** colorectal cancer, screening implementation, inequalities, lifestyle, Spain

## Abstract

*Background*: In Spain, colorectal cancer screening using the fecal occult blood test, targeted towards the 50–69 age bracket, was implemented on different dates. We aim to assess the temporal trend of colorectal cancer (CRC) screening uptake according to the year of screening implementation in each region and to identify predictors for the uptake of CRC screening. *Methods*: A cross-sectional study with 12,657 participants from the Spanish National Health Surveys 2011 and 2017 was used. Uptake rates were analyzed according to the date that the screening program was implemented. *Results*: For regions with programs implemented before 2011, the uptake rate increased 3.34-fold from 2011 to 2017 (9.8% vs. 32.7%; *p* < 0.001). For regions that implemented screening within the 2011–2016 period, the uptake rose from 4.3% to 13.2% (3.07-fold; *p* < 0.001), and for regions that implemented screening after 2016, the uptake increased from 3.4% to 8.8% (2.59-fold; *p* < 0.001). For the entire Spanish population, the uptake increased 3.21-fold (6.8% vs. 21.8%; *p* < 0.001). Positive predictors for uptake were older age, Spanish nationality, middle-to-high educational level, suffering chronic diseases, non-smoking and living in regions where screening programs were implemented earlier. *Conclusions*: The different periods for the implementation of CRC screening as well as sociodemographic and health inequalities may have limited the improvement in the screening uptake from 2011 to 2017 in Spain.

## 1. Introduction

Colorectal cancer (CRC) is considered a major public health problem with high incidence and mortality rates [1,2,3,4]. In Spain, according to data from GLOBOCAN 2018, 37,172 new cases of CRC were reported in 2018, reaching up to 13.7% of the total new cancer diagnoses. Furthermore, there were 16,577 deaths due to CRC (12.0 deaths per 100,000 habitants), the second-highest after lung cancer [5]. When diagnosed at an early stage, the five-year survival rate is around 94%; however, only one-fifth of CRC is diagnosed in an early stage [6]. 

Initiatives based on promoting a healthy diet and lifestyle and reducing exposure to recognized cancer risk factors are crucial primary intervention points for cancer prevention, which results in a reduction in CRC rates [7]. Cancer screening is considered a successful secondary prevention method to improve prognosis [8,9]. A CRC screening program was progressively implemented from 2000 onwards in Spain, with each region introducing the program on different dates [10]. This program was focused on male and female populations aged 50–69 years, and was conducted using a personal invitation letter every two years, which asked them to perform the fecal occult blood test (FOBT); when a positive result was returned, the person was instructed to have a colonoscopy performed [11,12,13]. The CRC screening program always used the same recruitment and sample collection methods, no matter the time of implementation.

European guidelines recommend at least 45% of the population to undergo FOBT to provide any benefit [10]; however, previous studies have reported that adherence for CRC screening was below 22% [14]. 

In our study, we aim to analyze the temporal trend in the adherence to CRC screening in people aged 50–69 years using data obtained from the Spanish National Health Surveys (SNHSs) conducted in 2011 and 2017. We assessed the uptake according to the year of implementation of the CRC screening in each Spanish region. We also analyzed possible sociodemographic, health and lifestyle-related variables that predict uptake of CRC screening.

## 2. Materials and Methods

### 2.1. Study Design

A cross-sectional study was performed based on data obtained from SNHSs 2011 and 2017. Both SNHSs were conducted by computer-assisted personal interviews at the participant’s households. The information collection period lasted for one year, from July 2011 to July 2012 for the SNHS 2011 and from October 2016 to October 2017 for the 2017 SNHS. The SNHS is a periodical survey that constitutes the main source of information on the perceived health of the population residing in Spain. It is performed every six years and is a part of the National Health System’s (NHS) Information System. The time that the data are collected, as well as the questions asked by the SNHS, are defined by the Ministry of Health, Social Services and Equality and the Spanish National Statistics Institute.

Tri-stage sampling was used, with stratification of the items in the first stage (census sections) having a probability proportional to the size of the section. The items in the second stage (principal family dwelling) were selected by systematic sampling with random start points and an equal probability for the selection of each family dwelling in the section. In the third stage, an adult (age ≥ 15 years) was selected to complete the questionnaire [15,16]. In the 2011 SNHS, 26,502 interviews were conducted, 21,007 of which were conducted among adults aged ≥ 15 years, while 29,196 interviews were conducted in the 2017 SNHS, with 23,090 among adults aged ≥ 15 years. More details on the methodology of SNHSs 2011 and 2017 are available in [15,16].

In the present study, according to the age recommendation for FOBT screening, we included 5688 and 6969 participants aged 50–69 from the 2011 and 2017 SNHSs, respectively.

### 2.2. Study Variables

The main study variable was the uptake of FOBT-based CRC screening. To identify those who received this screening test, we used the following questions included in the SNHSs questionnaires.

- ‘*Have you ever undergone a fecal occult blood test?*’. Those who answered affirmatively were asked a second question, ‘*When was the last time you had a fecal occult blood test?’* The following response options were given: “In the past 12 months”, “Over 1 year but less than 2 years ago”, “Over 2 years but less than 3 years ago”, “Over 3 years but less than 5 years ago”, “Over 5 years ago”. Subjects who reported that they had undergone FOBT within the previous two years were considered ‘uptake’, and the remaining subjects were classified ‘not-uptake’.

The independent variables considered included sociodemographic features variables, health status and lifestyle behaviors.

- Sociodemographic variables. Age, marital status (single/married/other), nationality (Spanish born/immigrant), education level (primary/secondary/university studies) and social class (upper/median/lower). Social class is based on occupation and created using the method proposed by the Spanish Society of Epidemiology [15,16].
- Health status. This included the number of self-reported chronic diseases. It was categorized into three groups according to the number of chronic diseases: “none”, “one or two” and finally “three or more” chronic diseases. The following chronic diseases were included to create this variable: high blood pressure, myocardial infarction and other heart diseases, asthma, emphysema, chronic bronchitis, chronic obstructive pulmonary disease, arthrosis, diabetes, cirrhosis, hepatic dysfunction, embolisms, ictus/stroke, malignancies and thyroid problems.
- Lifestyle behaviors. This included the following self-reported categories: current smoking, any alcohol consumption in the previous two weeks, leisure-time, physical activity and obesity (self-reported body mass index ≥ 30 kg/m^2^).

In each SNHS, participants were also classified according to the year in which the CRC screening program was implemented in their region. According to the date of implementation of the FOBT program, three groups were created: 

Implementation prior to 2011: participants residing in regions where CRC screening programs were implemented before 2011 (Regions: Canarias (2009), Cantabria (2008), Castilla y León (2010), Cataluña (2000), C. Valenciana (2005), Murcia (2005) and País Vasco (2009) and Rioja (2010)).

Implementation from 2011 to 2016: participants residing in regions where CRC screening programs were implemented between 2011 and 2016 (Regions: Andalucía (2014), Aragon (2013), Asturias (2014), Baleares (2015), Castilla la Mancha (2015), Galicia (2013) and Navarra (2013)).

Implementation after 2016 participants residing in regions where CRC screening programs had been not fully implemented by 2016 (Regions: Extremadura (2017), Madrid (2017) and Ceuta (2017) and Melilla (2017)).

### 2.3. Statistical Methods

The sample distribution of people was included in the CRC screening recommendations and according to the implementation date of the FOBT programs by regions. Adherence to FOBT was analyzed according to the independent study variables. 

Qualitative variables were expressed as frequencies and percentages. Comparisons were made using the chi-squared test. Multivariable analyses were performed using logistic regression, generating four models—one model for each of the three types of regions, according to the implementation date, and one with the entire sample to assess the effect of the moment of implementation in the uptake. Using these models, we can evaluate the adjusted change from 2011 to 2017 and the predictors of uptake for FOBT. The models included variables with a significant association in the bivariate analysis or reported as relevant in the literature. Statistical analysis was performed using the software SPSS 25.0 (IBM Corporation, Armonk, NY, USA). A *p*-value < 0.05 was considered as being statistically significant (two tails).

## 3. Results

### 3.1. Characteristics of the Target Populations for Fecal Occult Blood Test Screening

**Figure 1:**
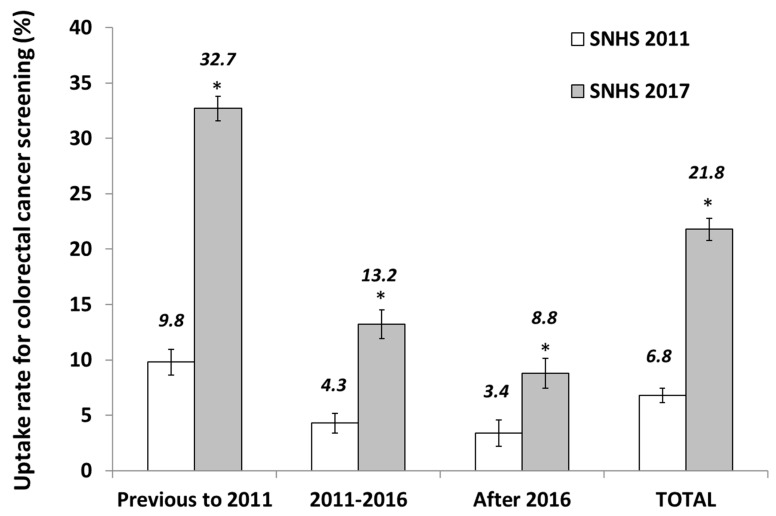
Uptake rates for colorectal cancer screening obtained from the 2011 and 2017 Spanish National Health Surveys (SNHSs) for people aged 50–69 years, according to the year the colorectal screening was implemented. Error bars represent a confidence interval of 95%. * *p*-value < 0.001 for the data from the Spanish National Health Survey 2011.

The study population included a total of 12,657 participants aged 50–59 years interviewed in the 2011 and 2017 SNHSs. In the 2011 SNHS, 5688 participants were analyzed. The distribution by study variables and according to the start-dates for the CRC programs were implemented as shown in Table 1 and Table 2. Table 1 shows that 2679 (47.10%) participants residing in regions with a FOBT program that was implemented before 2011. Among these residents, 9.8% reported undergoing a FOBT in the previous two years (see Figure 1). The equivalent data for residents in regions without screening programs or with an FOBT program implemented after 2016 were significantly lower (3.95%; 119/3009; *p* < 0.001).

There were 6969 people in the target population interviewed for the 2017 SNHS; their characteristics are shown in Table 2.

Figure 1 shows the FOBT uptake from the 2011 SNHS to the 2017 SNHS according to the date of implementation. The uptake among residents in regions with FOBT programs implemented before 2011 increased to 32.7% in the 2017 SNHS, this represents a 3.38-fold increase over time (*p* < 0.001). For regions that implemented the FOBT screening within the 2011 to 2016 period, the uptake rose from 4.3% to 13.2% (3.07-fold; *p* < 0.001), and for regions without FOBT programs by 2017, uptake increased from 3.4% to 8.8% (2.59-fold; *p* < 0.001). Figure 1 shows that for the entire Spanish population, there was a significant increase in the rate of FOBT uptake from 2011 to 2017 (6.8% vs. 21.8%, respectively; *p* < 0.001). 

### 3.2. Comparison of Uptake Rates for Fecal Occult Blood Test between 2011 SNHS and 2017 SNHS According to Sociodemographic Variables

Table 3 shows a significant increase in FOBT uptake from 2011 to 2017 in all categories of the sociodemographic variables analyzed and in the three types of regions according to the implementation date; however, the “single” category (marital status) in those communities where CRC screening had not been yet implemented by 2017 did not experience a similarly significant increase (3.5% vs. 4.6%; *p* = 0.568).

For the entire population, nationality was the only variable significantly associated with FOBT uptake in the 2011 SNHS, with higher uptake found in the Spain-born population. In the 2017 SNHS, the results indicate that age, nationality and social class were variables statistically associated with FOBT uptake, with a higher uptake among the Spain-born population, those aged 63–69 years and with higher social class (Table 3). Supplementary Figures S1–S5 show the distribution of FOBT uptake in the 2011 and 2017 SNSHs sorted by implementation date and according to gender (Supplementary Figures S1 and S2) and age groups (Supplementary Figures S3–S5). As Supplementary Figures S1 and S2 show, FOBT uptake in male and female follows a similar pattern in the SNHSs 2011 and 2017, revealing that the highest FOBT uptake was observed in Spanish regions where CRC screening programs were implemented before 2011 and it was smaller as CRC screening implementation was delayed. In this line, population grouped by range age (50–56, 57–62 and 63–69 years) also showed the highest FOBT uptake in Spanish regions where CRC screenings were implemented before 2011 and the worst FOBT uptake ratio in those regions where implementation was after 2016 (Supplementary Figures S3–S5).

### 3.3. Comparison of Uptake Rates for Fecal Occult Blood Test between SNHS 2011 and 2017 According to Health and Lifestyle-Related Variables

Table 4 shows a significant increase in the uptake from 2011 to 2017 in all the categories of health and lifestyle-related variables studied for the total population and those regions with FOBT implemented before 2011 or between 2011 and 2016. For the after 2017 regions, all categories increased significantly, except for subjects without chronic diseases, not-physically active and obese.

People with three or more chronic diseases showed higher adherence to FOBT screening than those with none, one or two chronic diseases in both 2011 and 2017 SNHSs. In the 2017 SNHS, participants that reported not smoking and practicing physical activity had significantly higher adherence to FOBT. 

### 3.4. Predictors for Fecal Occult Blood Test Uptake According to the Date of CRC Screening Programs Implementation

For the total population, we found that being aged 57–69, of Spanish nationality, having a secondary and university education, having one or more chronic diseases and being a non-smoker were positive predictors for FOBT uptake (Table 5). The results also reveal that for the 2017 SNHS, when compared to the 2011 SNHS, the probability of reporting uptake of FOBT was over four times more likely (OR 4.38 95%CI 3.87–4.96) (Table 5). 

To assess the effect of the implementation date, we used the ‘after 2017′ category as a reference and found that residents in regions implemented FOBT screening before 2011 had a 4.70-fold (95%CI 3.86–5.73) higher probability of reporting uptake, and those living in regions where FOBT implementation was between 2011 to 2016 had a 1.50-fold increase in probability (95%CI 1.21–1.85) (Table 5). 

Positive predictors of FOBT uptake were similar in regions that implemented programs before 2011 and the total population. 

Being aged 63–69, having a secondary and university study level and three or more chronic diseases were identified as positive predictors for FOBT uptake in regions where FOBT screening was implemented between 2011 and 2016. Interestingly, three or more chronic diseases was also identified as a positive predictor for FOBT uptake in regions where screening had not been established by 2017.

Finally, after multivariable adjustment, not including the FOBT implementation date, in all regions, there were significant improvements in uptake from 2011 to 2017; however, the greatest improvement was found in regions with programs starting before 2011 (OR 5.01; 95%CI 4.31–5.84) followed by those that began between 2011 and 2016 (OR 3.59; 95%CI 2.80–4.60) and the lowest increase (OR 2.69; 95%CI 1.75–4.14) for those living in regions without a program in 2017.

## 4. Discussion

In the present study, the temporal trend of uptake for CRC screening using FOBT was analyzed according to data from 2011 and 2017 SNHSs in the 50–69 years-old population. Our results show limited improvement in adherence to FOBT from 2011 to 2017 in Spain and that the delay in the date of CRC screening implementation seems to play a significant role in the low adherence to FOBTIn 2009, the Health, Social Services and Equality Ministry of Spain proposed a 100% uptake rate to FOBT in the 50–69 years-old population living in Spain by 2025 [17]; however, given our results, it seems unlikely. We found that the adherence to preventative FOBT screening increased from 6.8% in 2011 to 21.8% in 2017, representing a significant 15% increase. The adherence rate to FOBT found in the present work is much lower than the acceptable level of uptake participation rate of over 45% and the recommended level over 65% established by the European guidelines [18]. It is important to note that the low level of FOBT uptake was markedly influenced by the unequal CRC screening program implementation carried out in Spain. Regarding this point, regions where screening programs were implemented before 2010 reached 32.7% of FOBT adherence, whereas regions without a fully established CRC screening program by 2017 only reached an adherence of 9%.

It is difficult to make a precise comparison of the CRC screening adherence between the European Union countries since there are several differences among preventive screening programs in terms of update data, target age groups, screening interval and the primary test used in each country [19]; however, participation rates found in other European countries, with similar programs focused on the 50–69 year-old population, are markedly higher than the 21.8% found in the present work, e.g., Italy (54.4%) and Slovenia (60.4%) [20]. According to our data, Spain is among the European countries with the lowest participation rate, next to Croatia (19.9%) and the Czech Republic (22.7%) [20].

Identifying potential obstacles or inequities that may decrease adherence to FOBT is crucial [10]. In the present study, the possible relationship between sociodemographic and health variables and adherence to CRC screening were analyzed to identify the positive factors and barriers to FOBT uptake. 

Age was one of the most important predictors of screening uptake for CRC, with adherence to FOBT increasing with age. This finding is consistent with previous studies carried out in Spain and other countries [21,22,23]. A plausible explanation, since CRC incidence increases with age, is that the risk perception for developing CRC increases with age, resulting in a higher screening rate as people get older.

Additionally, being an immigrant was identified as a negative predictor for FOBT [24,25]. Language difficulties, embarrassment and even the expression of culturally influenced health beliefs have been reported in different countries as barriers for immigrants [26,27,28]. Interventions such as written and electronic communications adapted to geographic origin have proved useful to increase adherence to CRC screening among the immigrant population and should be implemented in our country [29,30]. 

Another remarkable finding was that a higher educational level was a positive predictor of uptake for FOBT. Higher educational level has been previously associated with higher use of preventive services and cancer screening rates, including CRC screening [14,31]. Knowledge of at least one CRC warning sign or symptom was an independent predictor for participation in CRC screening and was associated with using CRC procedures and being up to date with screening [32,33]. Another study reported that participants who knew that their own risk was higher than the average-risk population showed higher adherence to CRC screening (98%) than those who answered ‘same risk’ (84%) or ‘lower risk’ (74%) than the average population [34].

In our study, the number of reported chronic diseases was positively associated with adherence to CRC screening. Previous works also established a positive relationship between higher comorbidity and uptake for CRC screening, based on a higher frequency of visits to a health center and preoccupation with their health status [32,35]; however, it has also been suggested that the effect of specific diseases on CRC screening should be studied separately [36,37], the argument being that diseases with different severity, which require a different regimen of visits to health centers, should not be considered a part of the same variable. In our opinion, higher comorbidity may be positively associated with higher CRC uptake due to increased use of health services, since suffering chronic diseases is associated with regular check-ups. 

Regarding lifestyle variables, our results reveal that being a non-smoker was associated with a higher adherence to cancer screening. Several studies have shown that current smokers were less likely to receive or to be compliant with CRC screening [38,39,40]. As a possible explanation, health behavior choices may reflect an individual’s perceptions of the importance of overall good health, health risk prevention and perceived risk; however, we did not find a statistically significant association between other healthy lifestyles such as non-obesity or high physical activity and higher odds of screening. This absence of association could be due to a certain degree of distortion of the results commonly observed in self-reporting—perhaps subjects tend to under-report their body weights and over-report their heights and amount of time engaged in physical activity [41].

One of the most important findings of our work is identifying that the CRC screening implementation date was a very important positive predictor for FOBT adherence. Our results indicate that residents in regions that implemented CRC screening before 2011 had an almost five-fold higher probability of reporting uptake, whereas those living in regions that implemented screening between 2011 and 2016 had a 1.50-fold increase. Regions that established CRC screening early will have had more time and experience to solve specific organizational problems underlying low participation. A work performed in Italy one year after the implementation of CRC screening reported that one third of the target population who did not participate was due to administrative mistakes [42]. Spain is divided into 19 regions with a high level of self-government in different areas, including health service organization and provision. Although there is an Inter-Regional Coordination Board managed by the Spanish Health Ministry that shares and approves health issues, healthcare delivery is not identical. In this regard, the year of CRC screening implementation and even the analytical method of FOBT (Guaiac-FOBT or fecal immunochemical test (FIT) differed among regions over the study period. Interestingly, the European CRC screening program focused on a population between 50 and 69 years and with the highest rates of uptake for FOBT belonging to countries where CRC screening was simultaneously established across its region, such as Italy and Slovenia [43]. Although this comes with limitations and caution, it suggests that a nationwide CRC screening implementation in all Spanish regions could have induced higher participation. On the other hand, we have shown that regions in which CRC screening was implemented previously before 2011 had a higher uptake for FOBT than regions that established screening between 2011 to 2016. According to this finding, it would be plausible to think that information campaigns regarding CRC screening are not sufficient to raise awareness among the target population, and its effectiveness highly depends on the implementation period. The invitation model may also play an important role in low participation in CRC screening. In Spain, CRC screening programs are based on a single invitation letter every two years asking the subject to undergo FOBT, which they have to collect in pharmacies, and, if the test returns positive, a colonoscopy [11,44]. As a possible alternative, FOBT kits may be sent to the individuals’ homes to avoid having to collect the kit from another location. In this regard, Finland and the United Kingdom, countries with two of the highest uptake rates in Europe, carry out a CRC screening program based on the sending of an invitation letter, information on CRC screening, FOBT kits, instructions on how to perform the test and a prepaid return envelope [45,46]. On the other hand, several works have concluded that the first screening experience seems to play an important role, positively influencing adherence to FOBT; therefore, it would be crucial to ensure that the first participation is positive. Studies have shown that repeated invitations to CRC screening can successfully engage a substantial number of previous non-responders, providing information by post, email, telephone and on a face-to-face basis [47,48]. In addition, a lack of recommendation by health professionals may be considered a barrier to CRC screening participation [49].

Additional efforts should be made to reduce inequalities through performing new comprehensive health education campaigns to inform the general population and, particularly, to the target population about the high prevalence of CRC, its primary risk factors and prevention measures and the benefits of taking part in CRC screening programs [7,9,10]. Specific recommendations in the native language for the immigrant population should be performed in accordance with religious and cultural beliefs. It would be desirable to establish community organizations to ensure that screening information and strategies are culturally appropriate and relevant, monitoring those individuals who are considered hard-to-reach and have never been screened; this is particularly important since the largest impact from CRC screening is accrued from the first round. Additionally, novel mechanisms of collecting FOBT-kits may be also taken into consideration, e.g., sending FOBT-kit to individuals´ homes [45,46].

The representative at national and regional levels sample analyzed in the present study allowed us to determine the adherence to CRC screening as well as to establish predictors of FOBT uptake. Our results provide a pattern of performance and trends of CRC screening programs in Spain over the study period; however, there are also several study limitations. First, data from the SNHS may be affected by non-response bias, memory bias or the tendency of interviews to give socially desirable responses. Second, the type of analytical method of FOBT (Guaiac-FOBT or fecal immunochemical test (FIT) was not collected in the SNHSs. Finally, the use of a cross-sectional design means causality cannot be inferred, as “reverse causality” must be interpreted. Additionally, it is important to note that CRC has an important genetic/familial background. The heritable component of CRC is around 35% [50], with up to 3–5% of all CRC being hereditary [51]; however, in the SNHS from 2011 and 2017, there was no specific question about genetic/familial background; therefore, it was not possible to investigate heritability.

Beside these well-known limitations of health surveys, we consider that these databases are a valid source of information to determine the adherence to CRC screening as well as to establish predictors of FOBT uptake. Investigators from the US, Spain and other countries periodically have used health surveys to assess compliance and to identify uptake predictors for cancer screening program [14,25,31,52,53,54,55,56].

## 5. Conclusions

We conclude that the trend of FOBT adherence from 2011 to 2017 increased less than expected, suggesting that screening programs for CRC seem to be inadequate. Percentages of adherence rate for FOBT found in the 2011 and 2017 SNHSs are low. Based on the differences in the period of CRC screening implementation as well as sociodemographic variables, we suggest that health inequalities may have played a relevant role. Factors such as youth, immigration, lower educational level, not suffering from chronic conditions, smoking habits and living in regions with recent CRC screening implementation are negative predictors for screening uptake. 

## Figures and Tables

**Table 1:** Distribution of study populations for the fecal occult blood test (FOBT) screening by study variables according to the date colorectal cancer (CRC) programs were implemented. Results from the Spanish National Health Survey 2011.

		SPANISH NATIONAL HEALTH SURVEY 2011			
		Date of FOBT Screening Implementation			
Variables	Categories	Before 2011 (2679)	2011–2016 (2105)	After 2016 (904)	Total (5688)
		*N* (%)	*N* (%)	*N* (%)	*N* (%)
Gender	Male	1330 (49.6)	1037 (49.3)	445 (49.2)	2813 (49.4)
	Female	1349 (50.4)	1068 (50.7)	459 (50.8)	2876 (50.6)
Age groups ^a^	Low	1075 (40.1)	879 (41.8)	377 (41.7)	2331 (41.0)
	Middle	770 (28.7)	582 (27.6)	265 (29.3)	1617 (28.4)
	High	834 (31.1)	644 (30.6)	262 (29.0)	1740 (30.6)
Marital status	Married	2038 (76.1)	1641 (78.0)	710 (78.6)	4390 (77.2)
	Single	254 (9.5)	181 (8.6)	90 (10.0)	525 (9.2)
	Other	387 (14.4)	283 (13.4)	104 (11.4)	773 (13.6)
Nationality	Immigrant	197 (7.4)	128 (6.1)	60 (6.6)	384 (6.8)
	Spanish	2482 (92.6)	1977 (93.9)	844 (93.4)	5304 (93.2)
Educational level	Primary studies	589 (22.4)	711 (34.5)	178 (20.0)	1478 (26.5)
	Secondary studies	1690 (64.3)	1135 (55.2)	517 (58.1)	3342 (59.9)
	University studies	351 (13.3)	212 (10.3)	194 (21.8)	757 (13.6)
Social Class	Upper	520 (19.9)	368 (17.9)	231 (26.2)	1119 (20.2)
	Median	988 (37.8)	665 (32.4)	336 (38.2)	1989 (35.8)
	Lower	1107 (42.3)	1021 (49.7)	313 (35.6)	2441 (44.0)
Chronic diseases	0	979 (36.5)	719 (34.2)	366 (40.2)	2062 (36.2)
	1 or 2	1362 (50.8)	1096 (52.1)	436 (48.2)	2894 (50.9)
	3 or more	338 (12.6)	290 (13.8)	105 (11.6)	733 (12.9)
Smoker	Yes	651 (24.3)	517 (24.6)	208 (23.2)	1376 (24.3)
	No	2026 (75.7)	1586 (75.4)	685 (76.8)	4297 (75.7)
Alcohol Consumption	Yes	818 (68.6)	759 (74.9)	84 (18.5)	1945 (73.2)
	No	375 (31.4)	255 (25.1)	368 (81.5)	713 (26.8)
Physical Activity	Yes	1571 (58.7)	1001 (47.7)	558 (61.7)	3229 (56.9)
	No	1107 (41.3)	1100 (52.2)	343 (38.1)	2450 (43.1)
Obesity	Yes	1989 (74.2)	1362 (64.7)	683 (75.6)	1256 (29.1)
	No	558 (20.8)	524 (24.9)	174 (19.2)	4034 (70.9)

^a^ Age groups are as follows: low: 50–56 years; middle: 57–62 years; high: 63–69 years. FOBT: fecal occult blood test. “*N*” is referred to number of individuals (frequency) and “%” to percentage in each comparison group.

**Table 2:** Distribution of study populations for the fecal occult blood test screening by study variables according to the date CRC programs were implemented. Results from the Spanish National Health Survey 2017.

		SPANISH NATIONAL HEALTH SURVEY 2017			
		Date of FOBT Screening Implementation			
Variables	Categories	Before 2011 (3309)	2011–2016 (2573)	After 2016 (1087)	Total (6969)
		*N* (%)	*N* (%)	*N* (%)	*N* (%)
Gender	Male	1623 (49.0)	1277 (49.6)	521 (48.0)	3421 (49.1)
	Female	1686 (51.0)	1296 (50.4)	566 (52.0)	3547 (50.9)
Age groups ^a^	Low	1430 (43.2)	1061 (41.3)	474 (43.6)	2966 (42.6)
	Middle	905 (27.4)	752 (29.2)	328 (30.2)	1985 (28.5)
	High	973 (29.4)	759 (29.5)	285 (26.2)	2017 (28.9)
Marital status	Married	2542 (76.8)	2006 (78.0)	858 (78.9)	5407 (77.6)
	Single	324 (9.8)	214 (8.3)	100 (9.2)	638 (9.2)
	Other	443 (13.4)	352 (13.7)	129 (11.9)	924 (13.3)
Nationality	Immigrant	318 (9.6)	170 (6.6)	137 (12.6)	624 (9.0)
	Spanish	2991 (90.4)	2403 (93.4)	950 (87.4)	6344 (91.0)
Educational level	Primary studies	951 (29.1)	882 (34.9)	232 (21.5)	2065 (30.0)
	Secondary studies	1763 (53.8)	1273 (50.3)	572 (53.1)	3609 (52.4)
	University studies	566 (17.3)	374 (14.8)	273 (25.4)	1214 (17.6)
Social Class	Upper	625 (19.2)	406 (16.0)	274 (25.6)	1304 (19.0)
	Median	1166 (35.9)	884 (34.8)	383 (35.8)	2434 (35.5)
	Lower	1456 (44.8)	1252 (49.2)	413 (38.6)	3121 (45.5)
Chronic diseases	0	1373 (41.5)	933 (36.3)	456 (42.0)	2762 (39.6)
	1 or 2	1574 (47.6)	1296 (50.4)	519 (47.7)	3389 (48.6)
	3 or more	362 (10.9)	344 (13.3)	112 (10.3)	818 (11.7)
Smoker	Yes	880 (26.6)	667 (25.9)	264 (24.3)	1811 (26.0)
	No	2426 (73.3)	1904 (74.1)	823 (75.7)	5153 (74.0)
Alcohol Consumption	Yes	1002 (30.3)	786 (30.6)	344 (31.7)	4836 (69.4)
	No	2307 (69.7)	1786 (69.4)	743 (68.3)	2132 (30.6)
Physical Activity	Yes	2119 (36.0)	1655 (64.4)	386 (35.5)	4475 (64.2)
	No	1190 (36.0)	916 (35.6)	701 (64.5)	2492 (35.8)
Obesity	Yes	633 (19.6)	605 (24.3)	216 (20.4)	1455 (21.5)
	No	2591 (80.4)	1887 (75.7)	846 (79.6)	5324 (78.5)

^a^ Age groups are as follows: low: 50–56 years; middle: 57–62 years; high: 63–69 years. FOBT: fecal occult blood test. “*N*” is referred to number of individuals (frequency) and “%” to percentage in each comparison group.

**Table 3:** Uptake for the FOBT in the past two years among the target population by sociodemographic variables and according to the implementation date. Results from the Spanish National Health Surveys 2011 and 2017.

		Date of FOBT Screening Implementation							
		Before 2011		2011–2016		After 2016		TOTAL	
Variables	Categories	SNHS 2011 (2679)	SNHS 2017 (3309)	SNHS 2011 (2105)	SNHS 2017 (2573)	SNHS 2011 (904)	SNHS 2017 (1087)	SNHS 2011 (5688)	SNHS 2017 (6969)
		% (95% CI)	% (95% CI)	% (95% CI)	% (95% CI)	% (95% CI)	% (95% CI)	% (95% CI)	% (95% CI)
Gender	Male ^d,e,f,g^	10.0 (8.5–11.8)	32.3 (30.1–34.7)	4.1 (3.0–5.5)	14.1 (12.3–16.1)	3.2 (1.9–5.2)	9.3 (7.0–12.0)	6.7 (5.8–7.7)	22.0 (20.7–23.4)
	Female ^d,e,f,g^	9.5 (8.1–11.2)	33.1 (30.9–35.4)	4.6 (3.5–6.0)	12.3 (10.6–14.2)	3.5 (2.0–5.4)	8.2(6.2–10.7)	6.8 (5.9–7.7)	21.6 (20.2–22.9)
Age groups ^a,c^	Low ^d,e,f,g^	9.1 (7.4–10.9)	24.8 (22.6–27.1)	4.2 (3.0–5.7)	10.7 (9.0–12.8)	2.8 (1.4–4.8)	9.5 (7.1–12.5)	6.2 (5.3–7.3)	17.3 (16.0–18.7)
	Middle ^d,e,f,g^	9.0 (7.1–11.2)	38.7 (35.6–42.0)	4.3 (3.4–5.4)	13.6 (11.3–16.2)	3.6 (1.8–6.4)	8.4 (5.7–11.7)	6.4 (5.3–7.7)	24.2 (22.4–26.2)
	High ^d,e,f,g^	11.4 (9.4–13.7)	38.9 (35.8–42.0)	5.2 (2.9–8.1)	16.2 (13.7–18.9)	4.0 (2.1–6.9)	8.0 (5.4–11.8)	7.8 (6.6–9.1)	26.0 (24.1–28.0)
Marital status	Married ^d,e,f,g^	10.3 (9.1–11.7)	33.5 (31.7–35.3)	4.3 (3.4–5.4)	13.4 (12.0–15.0)	3.6 (2.4–5.3)	8.8 (7.0–10.8)	7.0 (6.3–7.8)	22.1 (21.1–23.3)
	Single ^d,e,g^	7.4 (4.8–11.3)	31.5 (26.5–36.6)	3.2 (1.1–6.4)	12.3 (8.3–17.1)	3.5 (0.9–8.6)	4.6 (2.0–10.7)	5.3 (3.6–7.5)	20.8 (17.8–24.1)
	Other ^d,e,f,g^	8.5 (5.9–11.5)	29.4 (25.3–33.8)	5.2 (2.9–8.1)	12.1 (9.1–16.0)	1.4 (0.1–4.5)	11.9 (7.0–18.0)	6.3 (4.7–8.2)	20.4 (17.9–23.1)
Nationality ^b,c^	Immigrant ^d,e,f,g^	5.3 (2.8–9.2)	12.0 (8.8–15.9)	2.4 (0.7–6.4)	9.5 (5.8–14.7)	0.0 (-)	10.0 (5.5–15.5)	3.5 (2.0–5.8)	10.9 (8.7–13.6)
	Spanish ^d,e,f,g^	10.1 (9.0–11.4)	34.9 (33.2–36.7)	4.5 (3.6–5.5)	13.4 (12.1–14.8)	3.6 (2.4–5.0)	8.6 (6.9–10.5)	7.0 (6.3–7.7)	22.9 (21.8–23.9)
Educational level	Primary ^d,e,f,g^	9.0 (6.9–11.6)	32.1 (29.1–35.1)	4.2 (2.9–5.8)	11.4 (9.4–13.6)	1.8 (0.5–4.6)	8.9 (5.6–12.9)	5.8 (4.7–7.1)	20.6 (19.0–22.5)
	Secondary ^d,e,f,g^	10.1 (8.7–11.6)	33.0 (30.9–35.3)	4.1 (3.1–5.4)	14.4 (12.5–16.4)	3.3 (1.9–5.0)	7.1 (5.2–9.4)	7.0 (6.2–7.9)	22.3 (21.0–23.7)
	University ^e,f,g^	10.5 (7.7–14.1)	33.4 (29.7–37.4)	4.3 (2.1–7.7)	14.2 (10.8–17.9)	5.2 (2.8–9.3)	12.2 (8.7–16.5)	7.4 (5.7–9.4)	22.7 (20.4–25.1)
Social Class ^c^	Upper ^d,e,f,g^	11.7 (9.2–14.7)	33.6 (29.9–37.3)	4.2 (2.4–6.6)	15.2 (11.9–18.8)	3.0 (1.2–5.6)	12.1 (8.7–16.4)	7.5 (6.1–9.2)	23.4 (21.2–25.8)
	Median ^d,e,f,g^	10.7 (8.9–12.8)	34.4 (31.8–37.2)	3.9 (2.7–5.7)	14.2 (12.0–16.6)	3.6 (2.0–6.2)	7.4 (5.1–10.3)	7.2 (6.2–8.5)	22.8 (21.2–24.5)
	Lower ^d,e,f,g^	8.3 (6.8–10.0)	31.7(29.3–34.1)	4.7 (3.5–6.1)	11.7 (10.1–13.6)	3.7 (1.9–6.1)	8.2 (5.7–11.0)	6.2 (5.3–7.2)	20.6 (19.2–22.0)

^a^ Age groups are as follows: low: 50–56 years; middle: 57–62 years; high: 63–69 years. ^b^ Significant association for the fecal occult blood test in the SNHS 2011. ^c^ Significant association for the fecal occult blood test in the SNHS 2017. ^d^ Significant difference of category distribution between the 2011 SNHS and the 2017 SNHS in the “before 2011” group. ^e^ Significant difference of category distribution between the 2011 SNHS and the 2017 SNHS in the “2011–2016” group. ^f^ Significant difference of category distribution between the 2011 SNHS and the 2017 SNHS in the “After 2017” group. ^g^ Significant difference of category distribution between the 2011 SNHS and the 2017 SNHS in the total population. CI: confidence interval.

**Table 4:** Uptake for the fecal occult blood test in the past two years among the target population by health status and lifestyle variables and according to the FOBT implementation date. Results from the Spanish National Health Surveys 2011 and 2017.

		Date of FOBT Screening Implementation							
		Before 2011		2011–2016		After 2016		TOTAL	
Variable	Categories	SNHS 2011 (2679)	SNHS 2017 (3309)	SNHS 2011 (2105)	SNHS 2017 (2573)	SNHS 2011 (904)	SNHS 2017 (1087)	SNHS 2011 (5688)	SNHS 2017 (6969)
		% (95% CI)	% (95% CI)	% (95% CI)	% (95% CI)	% (95% CI)	% (95% CI)	% (95% CI)	% (95% CI)
Chronic diseases ^a,b^	0 ^c,d,f^	6.0 (4.6–7.6)	27.7 (25.3–30.1)	2.5 (1.4–3.7)	11.7 (9.8–13.9)	3.8 (2.1–6.1)	5.5 (3.7–7.9)	4.4 (3.6–5.3)	18.6 (17.2–20.1)
	1 or 2 ^c,d,e,f^	11.5 (9.9–13.4)	36.4 (34.1–38.9)	4.3 (3.2–5.6)	13.2 (11.4–15.1)	3.4 (1.9–5.4)	11.8 (9.1–14.7)	7.6 (6.6–8.6)	23.8 (22.4–25.2)
	3 or more ^c,d,e,f^	13.7 (10.3–17.7)	35.8 (31.0–40.9)	9.3 (6.3–13.2)	17.2 (13.5–21.5)	1.8 (0.4–6.1)	8.1 (4.2–14.7)	10.3 (8.3–12.7)	24.2 (21.4–27.3)
Smoker ^b^	Yes ^c,d,e,f^	7.7 (5.8–9.9)	25.7 (22–9-28.7)	5.7 (3.9–7.9)	10.4 (8.2–12.9)	1.5 (0.4–3.9)	7.2(4.6–10.9)	6.0 (4.8–7.4)	17.4 (15.7–19.2)
	No ^c,d,e,f^	10.5 (9.2–11.9)	35.3 (33.5–37.3)	3.9 (3.0–5.0)	14.1 (12.6–15.8)	3.9 (2.6–5.6)	9.3 (7.4–11.3)	7.0 (6.3–7.8)	23.4 (22.2–24.5)
Alcohol Consumption	Yes ^c,d,e,f^	11.6 (8.5–15.0)	32.9 (31.0–34.9)	3.8 (2.1–7.0)	13.1 (11.6–14.7)	0.6 (0.1–5.4)	9.1 (7.2–11.3)	7.5 (5.8–9.7)	22.0 (19.7–23.2)
	No ^c,d,e,f^	8.9 (7.1–11.1)	32.3 (29.4–35.2)	4.3 (3.0–5.0)	13.3 (11.0–15.8)	3.8 (2.3–6.3)	8.0 (5.5–11.2)	6.2 (5.1–7.3)	21.4 (20.8–23.1)
Physical Activity ^b^	Yes ^c,d,e,f^	10.8 (9.3–12.4)	34.3 (32.3–36.4)	3.0 (2.1–4.1)	14.5 (12.9–16.3)	2.9 (1.7–4.5)	9.5 (7.5–11.8)	6.7 (5.8–7.8)	23.1 (21.9–24.4)
	No ^c,d,f^	8.3 (6.7–10.0)	30.0 (27.4–32.6)	5.9 (4.5–7.5)	10.7 (8.9–12.9)	4.2 (2.4–6.7)	7.4(5.1–10.3)	6.8 (6.0–7.7)	19.4 (17.9–21.0)
Obesity	Yes ^c,d,f^	11.6 (9.1–14.4)	33.3 (29.8–37.1)	4.7 (3.1–6.8)	13.7 (11.2–16.7)	3.7 (1.5–7.3)	8.1 (4.9–12.1)	6.3 (5.6–7.1)	22.2 (21.0–23.3)
	No ^c,d,e,f^	9.3 (8.1–10.7)	33.1 (31.3–34.9)	3.5 (2.6–4.6)	13.0 (11.6–14.6)	3.2 (2.0–4.7)	9.0(7.3–11.2)	7.7 (6.2–9.2)	21.4 (19.4–23.6)

^a^ Significant association for fecal occult blood test in the SNHS 2011. ^b^ Significant association for the fecal occult blood test in the 2017 SNHS. ^c^ Significant difference of category distribution between the 2011 SNHS and the 2017 SNHS in the “Before 2011” group. ^d^ Significant difference of category distribution between the 2011 SNHS and the 2017 SNHS in the “2011–2016” group. ^e^ Significant difference of category distribution between the 2011 SNHS and the 2017 SNHS in the “After 2017” group. ^f^ Significant difference of category distribution between the 2011 SNHS and the 2017 SNHS in the total population. CI: confidence interval.

**Table 5:** Variables independently and significantly associated with fecal occult blood test uptake after multivariable logistic regression analysis according to the date colorectal cancer screening was implemented. Results from the Spanish National Health Surveys 2011 and 2017.

		Date of FOBT Screening Implementation			
		Before 2011	2011–2016	After 2016	Total
Variables	Categories	OR (95% CI)	OR (95% CI)	OR (95% CI)	OR (95% CI)
Age groups ^a^	Low	1	1	1	1
	Middle	1.54 (1.31–1.82)	NS	NS	1.39 (1.22–1.58)
	High	1.59 (1.35–1.89)	1.42 (1.09–1.85)	NS	1.47 (1.28–1.68)
Nationality	Immigrant	0.30 (0.21–0.42)	NS	NS	0.42 (0.32–0.54)
	Spanish	1	1	1	1
Educational level	Primary studies	1	1	1	1
	Secondary studies	1.33 (1.13–1.56)	1.45 (1.14–1.84)	NS	1.31 (1.15–1.49)
	University studies	1.41 (1.15–1.74)	1.47 (1.06–2.06)	NS	1.47 (1.28–1.68)
Chronic diseases	None	1	1	1	1
	1 or 2	1.47 (1.27–1.70)	NS	NS	1.42 (1.26–1.59)
	3 or more	1.48 (1.18–1.84)	1.88 (1.38–2.58)	1.87 (1.24–2.82)	1.58 (1.33–1.89)
Smoker	Yes	1	1	1	1
	No	1.44 (1.23–1.68)	NS	NS	1.34 (1.18–1.52)
Year of SNHS	2011	1	1	1	1
	2017	5.01 (4.31–5.84)	3.59 (2.80–4.60)	2.69 (1.75–4.14)	4.38 (3.87–4.96)
CRC screening implementation	Before 2011	NA	NA	NA	4.70 (3.86–5.73)
	2011–2016	NA	NA	NA	1.50 (1.21–1.85)
	After 2017	NA	NA	NA	1

^a^ Age groups are as follows: low: 50–56 years; middle: 57–62 years; high: 63–69 years. CRC: colorectal cancer. SNHS: Spanish National Health Surveys. FOBT: fecal occult blood test; OR: odds ratio; CI: confidence interval; NS: not significant; NA: not applicable.

## Supplementary Materials

The following are available online at https://www.mdpi.com/1660-4601/17/17/6222/s1, Figure S1: Fecal occult blood test uptake from the SNHS 2011 to the SNHS 2017 among men and according to the date populations screening programs were implemented, Figure S2: Fecal occult blood test uptake from the SNHS 2011 to the SNHS 2017 among women and according to the date populations screening programs were implemented, Figure S3: Fecal occult blood test uptake from the SNHS2011 to the SNHS 2017 in population aged 50–56 years according to the date populations screening programs were implemented, Figure S4: Fecal occult blood test uptake from the SNHS 2011 to the SNHS 2017 in population aged 57–62 years according to the date populations screening programs were implemented, Figure S5: Fecal occult blood test uptake from the SNHS 2011 to the SNHS 2017 in population aged 63–69 years according to the date populations screening programs were implemented.
