# Supplementary material for: Trends and Predictors for the Uptake of Colon Cancer Screening Using the Fecal Occult Blood Test in Spain from 2011 to 2017
Source: Int J Environ Res Public Health. 2020 Aug 27;17(17):6222. doi: 10.3390/ijerph17176222 (PMC7504712; doi:10.3390/ijerph17176222)

Supplementary figure 1 . Fecal occult blood test uptake from the SNHS 2011 to the SNHS 2017 among men and according to the date populations screening programs were implemented

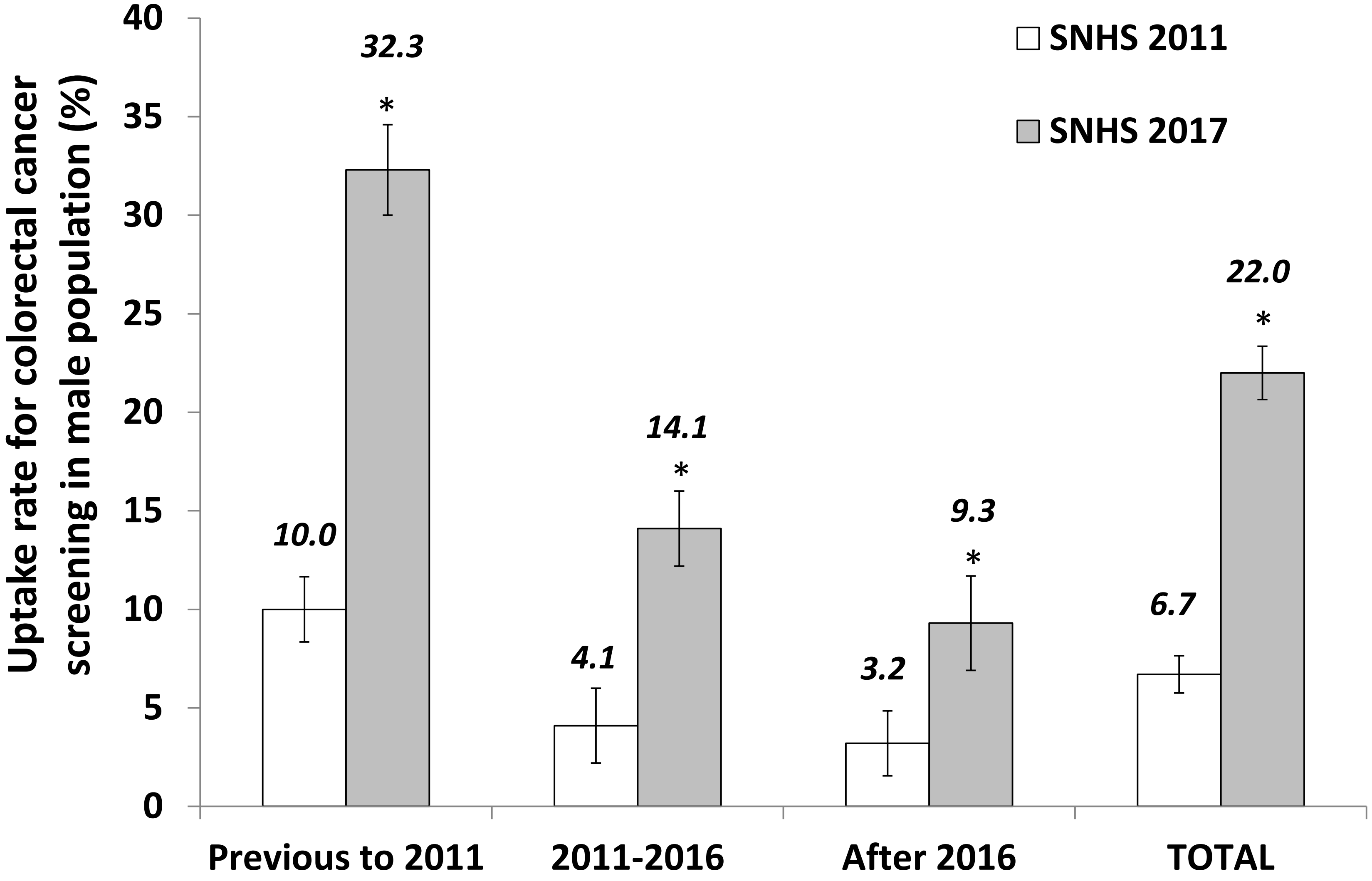

Supplement: Supplementary file 1 [file ijerph-17-06222-s001.zip › Supplementary Figure 1.pdf]
